# Shifting cultures of silence: a knowledge-integrated learning approach to organisational responsiveness on alcohol prevention - a longitudinal qualitative process study in Swedish workplaces

**DOI:** 10.1186/s12889-025-26009-5

**Published:** 2025-12-20

**Authors:** E. Wikström, M. Sager, M. Bertilsson, G. Hensing, K. Berglund, U. Hermansson, N. Gillberg

**Affiliations:** 1 Department of Business administration, School of Business, Economics and Law at the University of Gothenburg, Box 610, Göteborg, SE-40530 Sweden; 2 Department of Philosophy, Linguistics and Theory of Science, Faculty of Humanities at the University of Gothenburg, Göteborg, Sweden; 3 School of Public Health and Community Medicine, Sahlgrenska Academy at the University of Gothenburg, Göteborg, Sweden; 4 Department of Psychology, Faculty of Social Sciences at the University of Gothenburg, Göteborg, Sweden; 5 Department of Clinical Neuroscience, Centre for Psychiatry Research, Karolinska Institutet, Stockholm, Sweden

**Keywords:** Workplace health, Alcohol prevention, Organisational learning, Human resource practices, Organisational culture

## Abstract

**Background:**

Organisational responses to alcohol-related risks in the workplace are often constrained by cultural norms, emotional discomfort, and unclear preventive responsibilities. Although formal alcohol policies are common, their implementation is hindered by stigma and silence. This study examines how a knowledge-integrated learning process can support Human Resource (HR) professionals in reducing organisational tolerance for risky drinking and strengthening preventive responsiveness.

**Methods:**

A longitudinal qualitative intervention was conducted between 2022 and 2025 across seven large organisations in southwest Sweden. Fourteen HR professionals participated in three facilitated Learning Labs designed as dialogical arenas for knowledge input, reflection, and collaborative problem-solving. Data sources included a web survey of 5,868 managers, observational field notes, and transcripts from the Learning Labs. Reflexive thematic analysis was used to explore how HR participants’ understanding of alcohol prevention developed over time.

**Results:**

Five mechanisms shaped the learning process: dialogical co-created learning, experience-based reflection, emotional and cultural barriers, structural ambiguity and unclear mandates, and managerial insecurity. These mechanisms evolved across three phases: (1) emotionally grounded reflection on cultural silence, (2) conceptual reframing of preventive responsibility, and (3) method-driven application through tools and routines. Over time, HR participants shifted from reactive uncertainty to more proactive and structurally informed approaches to alcohol prevention.

**Conclusions:**

The study shows how sustained, participatory learning can enhance HR professionals’ preventive reasoning and ability to interpret alcohol-related risks. Integrating emotional, cultural, and structural dimensions enables a move beyond compliance-focused policy work toward more coherent and responsive preventive reasoning and approaches. The findings highlight the value of dialogical, culturally attuned, and structurally supported learning processes in strengthening workplace alcohol prevention.

## Background

 Alcohol consumption poses a global health challenge with profound economic, social, and public health implications. In many European and Nordic workplaces, alcohol consumption during working hours has largely been eliminated, particularly in knowledge-based sectors. Recent Danish research notes this as a significant public health achievement, alongside declining national alcohol consumption and shifting attitudes [22]. Nevertheless, alcohol-related risks persist across sectors because consumption during work hours and alcohol use outside work that affects performance or behaviour at work continues to affect performance, safety, absenteeism and productivity, and some industries, especially male-dominated sectors such as construction, show particularly high levels of hazardous drinking due to entrenched social norms [12, 23].

Addressing these behaviours requires multifaceted approaches that integrate individual, organisational, and cultural dimensions. While workplace interventions have gained traction, the interplay between organisational culture, alcohol norms, and employees’ everyday behaviours remains insufficiently understood, limiting the effectiveness of prevention strategies. Research from Nordic leadership and HRM contexts shows that organisational change is shaped not only by formal systems but also by implicit cultural values and emotionally grounded leadership ideals [14, 16, 17]. HR practitioners play a crucial yet understudied role in shaping how preventive responsibility is interpreted, enacted, or avoided in daily practice. At the same time, workplace alcohol issues are increasingly recognised as an emerging area within organisational health research, reflecting a broader shift toward understanding alcohol-related risks not merely as individual behaviours but as organisational phenomena linked to culture, leadership, and preventive readiness.

Despite policy developments, alcohol misuse remains embedded in workplace cultures, complicating implementation [1]. A persistent gap exists between written alcohol policies and everyday practice, often driven by stigma, uncertainty, and unclear mandates. To bridge this gap, prevention models increasingly emphasise participatory learning and culture-oriented approaches.

These challenges align with insights from complex systems research, which highlights that alcohol-related harms emerge from dynamic interactions across individual, organisational, and societal levels, requiring interventions that address interconnected mechanisms rather than isolated risk factors [21]. Within workplace health promotion, multi-component and adaptive intervention strategies, combining education, behavioural support, and policy frameworks, are shown to reduce tolerance for misuse and strengthen organisational responsiveness [1, 7].

Lessons from workplace mental health interventions further underline the value of participatory and learning-oriented approaches. Tailored problem-solving interventions can enhance engagement across organisational levels [3], while shared learning and co-creation processes strengthen the sustainability of workplace change efforts [10, 19]. Effective implementation also depends on organisational readiness, leadership engagement, and alignment with cultural norms [7]. Integrated frameworks bridging safety, health, and well-being offer useful guidance for designing alcohol prevention strategies [24].

In Sweden, national survey data (Health on Equal Terms 2024) show that 18% of men and 13% of women report hazardous consumption [11], reinforcing the relevance of workplace prevention efforts. While multi-component programs combining policy enforcement, education, and supervisor support have demonstrated promise [8], their success depends on understanding how workplace culture and HR actors shape tolerance and responsiveness in practice.

By drawing on multi-component intervention frameworks and integrating lessons from broader workplace health initiatives, this study addresses the following question: How can knowledge-integrated process learning models for alcohol prevention programs be designed, and perceived and operationalised by HR professionals, to reduce tolerance for risky drinking and enhance preventive responsiveness to alcohol-related issues?

### Toward a knowledge-integrated approach

Workplace alcohol prevention programs have evolved from strict, zero-tolerance policies toward more holistic and inclusive models. Early punitive approaches often faced resistance in industries with deeply ingrained social drinking norms [4]. More recent research emphasises integrating educational, behavioural, and policy-driven components into comprehensive prevention strategies [15], reflecting a growing recognition of the role that organisational culture, leadership, and peer dynamics play in shaping workplace behaviours.

As Sorensen et al. note, integrated frameworks for workplace safety, health, and well-being provide a strong foundation for designing prevention programs that are both adaptive and participatory [24]. Supervisor training has emerged as a key component: Elling et al. show that supervisor engagement increases responsiveness and leads to more timely interventions [9]. Participatory approaches, such as those described by Lalani et al., further demonstrate how co-created solutions enhance contextual relevance and acceptance [19].

A growing body of literature highlights the importance of addressing both individual behaviours and organisational cultures. Interventions targeting social and organisational norms, alongside individual behaviours tend to be more effective [7, 24]. Within this literature, HR practitioners increasingly appear as central actors, mediating how preventive policies and knowledge inputs are interpreted and enacted in practice. Yet, their perspectives remain underexplored, particularly in relation to tolerance, responsiveness, and organisational learning.

Drawing on insights from the multi-component model proposed by Elling et al. [8] and broader workplace health intervention research, this study highlights the importance of integrating collective learning, structured knowledge input, and policy enforcement into comprehensive prevention programs. By emphasising tolerance reduction and organisational responsiveness, the model addresses the interplay between individual behaviours and organisational culture. We conceptualise these dynamics at three interconnected levels: the ‘individual level’ (HR professionals’ reflections and interpretations), the ‘managerial level’ (HR’s perceptions of managerial engagement), and the ‘organisational level’ (structural, cultural, and policy-related conditions). Because the data derive from HR representatives, the study captures both their personal experiences and their observations of organisational practices. This perspective also helps identify key mechanisms through which knowledge dissemination and participatory learning may strengthen preventive capacity in workplaces.

### Tolerance and responsiveness in alcohol prevention

In workplace alcohol prevention, the concepts of tolerance and responsiveness form a crucial foundation for understanding how organisations manage risk. Tolerance refers to the extent to which organisations, formally or informally, accept or overlook risky drinking behaviours, not as endorsement but as an indication of where action thresholds lie. Responsiveness describes the organisation’s ability to act proactively and adaptively when faced with alcohol-related risks, through both reactive measures (e.g., interventions after incidents) and proactive strategies (e.g., early detection, education, and support).

These perspectives are deeply intertwined with organisational culture and industry-specific contexts. For example, in sectors such as hospitality and construction, alcohol use may be normalised, complicating the implementation of preventive policies [13]. Research shows that organisations striking a balance, reducing tolerance while increasing responsiveness, achieve better outcomes in reducing misuse [18].

Frone et al. found that workplaces combining clear alcohol policies with education and early interventions saw significant reductions in absenteeism and productivity loss [13]. This underscores the importance of integrated approaches that move beyond punitive measures toward cultures of awareness and accountability. Responsiveness as an organisational trait involves comprehensive policies, access to Employee Assistance Programs (EAPs), and equipping supervisors with tools to recognise and respond to risk [12]. Participatory approaches and shared learning further strengthen responsiveness by fostering collaboration between employees, HR, and leadership [19]. Daniels et al. stress that such approaches must be embedded in workplace culture and aligned with organisational norms [7]. Culturally grounded, multi-component strategies consistently yield more sustainable behavioural change than isolated or disciplinary actions [15].

### Learning-focused interventions

Learning-based interventions have become an essential part of workplace alcohol prevention. These initiatives promote awareness, responsibility, and long-term behaviour change through workshops, campaigns, and peer-led discussions [20]. Sorensen et al. argue that such efforts are most effective when embedded within broader safety and health frameworks [24]. Organisational learning theory supports this view: interventions aligned with organisational values and developed collaboratively tend to generate stronger engagement and lasting impact [19]. Participatory evaluation ensures that strategies address both individual behaviour and social dynamics within workplace culture [2].

Antonacopoulou’s concept of practising describes learning as an iterative process involving reflection, experimentation, and unlearning - an active engagement with tensions that helps link knowledge to meaningful goals [2]. Educational initiatives embedded within multi-component programs have been shown to reduce hazardous drinking, particularly when supported by feedback and iterative learning opportunities [9]. Daniels et al. similarly highlight that successful implementation requires organisational readiness, leadership commitment, employee involvement, and continuous adaptation [7].

In sum, tolerance and responsiveness are dynamic and interdependent elements that underpin effective workplace alcohol prevention. When combined with structured, learning-focused interventions, they form a comprehensive and sustainable strategy. Building on insights from Arends et al. [3], Daniels et al. [7], Lalani et al. [19], and Sorensen et al. [24], this study introduces a knowledge-integrated model grounded in participatory evaluation, supervisor engagement, and multi-level intervention, designed to reduce harmful tolerance, strengthen organisational responsiveness, and foster a culture of shared learning and continuous improvement.

## Methods

This study forms part of the larger ‘XXXX’ (anonymised) project, which investigates organisational and managerial conditions for alcohol-preventive actions in Swedish workplaces. While the broader project includes several components, the present article focuses specifically on the knowledge-integrated learning process model tested through a series of facilitated Learning Labs. The core elements of this intervention model are summarised in Table 1.

**Table 1:** Knowledge-integrated process model

Component	Description
Theoretical basis	Dialogue theory; drinking climate; organisational culture; learning; leadership
Facilitation	Three Learning Labs; participatory dialogue; collective inquiry; adaptive design
Knowledge input	Survey findings as structured knowledge input
Implementation	Seven organisations; 14 h professionals; three labs over two years
Outcome focus	Knowledge dissemination; reflection; strengthened preventive capacity

### Design, setting and participants

A prospective, longitudinal qualitative design (2022–2025) was used to explore how a knowledge-integrated learning process can strengthen alcohol-preventive work in organisations. The design combined a large manager survey with qualitative observations from three interactive Learning Labs. To clarify the structure of the study, Table 2 provides an overview of the methodological components, data sources, and intervention format.

**Table 2:** Overview of study design

Component	Description
Aim	Explore knowledge-integrated learning processes for prevention
Design	Longitudinal qualitative study with embedded quantitative elements
Participants	Seven organisations; 14 h professionals
Data sources	Survey; Learning Lab observations; field notes; transcripts
Duration	Three Learning Labs over two years
Intervention	Participatory dialogue; knowledge input; collaborative mapping
Analysis	Reflexive thematic analysis (Braun & Clarke)

The study involved seven large public and private organisations in southwest Sweden. Each organisation appointed two Human Resource professionals to participate, resulting in 14 h representatives. To increase transparency regarding the participant sample, a detailed overview is provided in Table 3.

**Table 3:** Participant characteristics

Organisation	Sector	HR Role	Participants
Org 1	Public	HR Partner	2
Org 2	Public	HR Specialist	2
Org 3	Private	HR Manager	2
Org 4	Private	HR Generalist	2
Org 5	Public	HR Consultant	2
Org 6	Private	HR Partner	2
Org 7	Public	HR Strategist	1–2

### Data collection

The study spans three years. Year 1 involved a comprehensive web-survey directed toward managers; Years 2–3 included three facilitated Learning Labs with HR representatives. The results from the survey functioned as structured knowledge input and played a central role in shaping discussions during all Learning Labs.

#### Structured knowledge input: the manager survey

The survey was distributed in late 2023 to 9,000 managers through a national sampling panel, yielding 5,868 responses (4,723 included in analysis). The survey comprised three parts:

1. Individual managerial factors (gender, age, education, tenure, attitudes toward alcohol prevention, personal experience).
2. Workplace contextual factors (industry, size, psychosocial environment, support from management/HR/OHS, alcohol norms).
3. Preventive measures (universal and selective efforts, perceived effectiveness, confidence in addressing risky use).

The survey revealed a notable implementation gap: although most managers believed employers carry preventive responsibility, only a minority reported taking any preventive action. Gender and sector differences were prominent. These contrasts became a key starting point for reflection in Learning Lab 1.

#### Learning labs and observation study

The Learning Labs served as dialogical arenas designed to integrate research-based knowledge with participants’ lived organisational experience. Three complementary knowledge bases informed the Labs:

1. survey results,
2. research literature, and.
3. participants’ own workplace experiences.

To structure reflection and make cognitive models visible, the Labs used a Two-Stage Conversational Mapping Approach, consisting of three progressive phases:

#### Stage 1 - initial exploration and mapping

Participants discussed why they intervene, or hesitate to intervene, when observing early signs of alcohol-related problems. Open-ended questions encouraged articulation of reasoning, dilemmas, and perceptions. Field notes were translated into preliminary node-link maps.

#### Stage 2 - showcasing and synthesising

In Learning Lab 2, researchers presented the maps created from Lab 1, which enabled deeper scrutiny of links between assumptions, norms, and organisational conditions. This phase helped reveal implicit cognitive models related to tolerance and preventive responsibility.

#### Stage 3 - potential, contribution, and outcome

Learning Lab 3 explored the perceived impact of the model and participants’ insights on organisational learning, dissemination, and preventive readiness.

### Data analysis

The analysis followed a reflexive thematic analysis approach inspired by Braun and Clarke [6]. The process involved:

1. Repeated readings of transcripts and field notes.
2. Open coding of meaning-bearing segments.
3. Grouping codes into preliminary themes.
4. Iterative refinement through team discussions.
5. Synthesising themes into overarching analytical perspectives aligned with the three Learning Labs.

Central themes included:

- Collective learning and reflection.
- Emotional and cultural barriers.
- Uncertainty about mandates.
- Implementation of preventive tools.

To increase analytic transparency, Table 4 presents the coding frame with themes, sub-themes, and illustrative quotes.

**Table 4:** Coding frame

Theme	Sub-theme	Description	Illustrative Quote	Lab
Dialogue-based learning	Collective reflection	Learning emerging through dialogue	“We just ask a question…”	Lab 1–3
Experience-based learning	Practical dilemmas	Emotional cases trigger learning	“A real employee case…”	Lab 1
Cultural barriers	Stigma & silence	Discomfort prevents intervention	“From snitching to caring”	Lab 1–2
Mandate uncertainty	Unclear roles	Ambiguity in responsibility	“Should we even work with risky use?”	Lab 2
Managerial insecurity	Need for tools	Supervisors insecure about action	“Managers are very insecure…”	Lab 3

In addition, Table 5 summarises how the five analytical mechanisms unfolded across the three Learning Labs, aligning the process with the emergent learning trajectory.

**Table 5:** Summary of findings across learning labs

Mechanism	Lab 1	Lab 2	Lab 3
Dialogue-based learning	Dialogue for early signals	Theoretical reframing	Proactive tool use
Experience-based reflection	Emotional cases	Policy–practice links	Applied practice
Cultural barriers	Stigma	Middle ground identified	Normalisation
Structural ambiguity	Reactive routines	Mandates questioned	Tools embedded
Managerial insecurity	Hesitation	Leadership framing	Confidence via tools

### Ethical considerations

The study was approved by the Swedish Ethics Review Board (Ref. No. 2023–06820-01). Participants received written and oral information and provided informed consent in line with the Helsinki Declaration’s principles of autonomy, non-maleficence, beneficence, and justice.

## Results

The findings of this study are based on a longitudinal analysis of data from three interactive Learning Labs. The Learning Labs brought together HR professionals from various organisations to collaboratively explore how alcohol-related issues are addressed in practice. These labs functioned as dialogical arenas for knowledge exchange and collective reflection, fostering shared learning across organisational boundaries. Rather than simply exchanging experiences, the labs initiated a deeper process of shared meaning-making, grounded in reflective dialogue and emotionally resonant learning. Learning was sparked not only by information but by interaction, emotion, and lived experience.

The analysis revealed five central mechanisms that shaped how participants developed preventive awareness and action:

1. Dialogue-based co-created learning,
2. Experience-based reflection and practical dilemmas,
3. Emotional and cultural barriers to action,
4. Structural ambiguity and unclear mandates, and.
5. Managerial insecurity and the importance of leadership support.

These mechanisms evolved through a three-phase process: beginning with emotionally charged reflections (Lab 1), advancing to theoretical understanding and strategic questioning (Lab 2), and culminating in applied, method-driven practice (Lab 3).

In the sections that follow, we present the results in alignment with this progression, illustrating how learning unfolded and how key mechanisms interacted to support a shift from reactive uncertainty to proactive and structurally supported responsiveness.

### Learning lab 1: building the foundations for collective and reflective learning

#### The role of the survey as structured knowledge input

Drawing on the results of the survey that initiated the research project’s data collection - which revealed a generally positive attitude toward preventive measures despite limited implementation - the following question was posed to the participants to initiate the discussion: *What does the gap between expressed positive attitudes and actual action indicate?*

#### Early signals and the role of dialogue in responsiveness

The first learning lab revealed how challenging it is to detect and interpret early warning signs of alcohol-related problems, particularly when signals are subtle or obscured. One participant explained, “Our structured work is generally about catching early signals…,” while another noted, “One challenge is that many work from home… it’s harder to see early signals.” These reflections illustrate the increasing fragility of responsiveness in digitally mediated workplaces, where reduced visibility can hinder early intervention. Conversation emerged as the primary tool for addressing these uncertainties. Rather than relying solely on formal mechanisms, participants stressed the importance of relational attentiveness, being able to sense, inquire, and respond through dialogue.

#### Cultural barriers - from ‘snitching’ to caring

A central theme in Learning Lab 1 was the cultural stigma that surrounds addressing alcohol use at work. Participants described a deep-seated discomfort, sometimes rooted in fear of being perceived as disloyal or accusatory. One participant asked: “How do you create change… from snitching to caring?” This question encapsulates the shift needed, from a punitive framing to one rooted in collective well-being. Even in organisations with formal policies, implementation was uneven. There was a shared recognition that without cultural support, policy frameworks often remain symbolic or underutilised. One participant remarked, “We enforce zero tolerance but face issues like employees arriving slightly hungover. Are they fit for work?” This illustrates the dissonance between written policy and lived reality.

#### Structural support, leadership, and sustainability

Participants frequently highlighted how alcohol prevention efforts are vulnerable when they depend on individual champions. “For it to work, the issue needs to stay current all the time,” one noted, expressing concern over the short-termism often present in prevention efforts. Another added, “Knowledge, interest, and priorities determine whether you work on it…,” suggesting that motivation is too often personalised rather than institutionalised. However, there was also evidence of a shift. “Today, it’s not in the person, it’s in the structure,” said one participant, signalling a growing understanding that sustainable alcohol prevention requires embedded routines, not just motivated individuals. External collaborations, such as with expert organisations on alcohol use within workplaces or occupational health services were also seen as critical in bolstering internal credibility and maintaining continuity, especially during leadership turnover or organisational change.

#### Experience, problem-solving, and learning through dilemma

Learning in the first lab unfolded across multiple forms: experiential, dialogical, and problem-oriented. Real cases prompted organisations to revise policies and practices. One participant explained, “A real employee case revealed inadequate routines, leading us to collaborate with x (expert organisation on alcohol use within workplaces) and update guidelines for employees and students.” These cases often involved emotionally and ethically complex dilemmas. Participants described being caught between confidentiality and responsibility: “Employees confide in managers who become constrained, struggling with confidentiality.” Another noted, “It’s challenging when individuals deny addiction diagnoses; we sometimes proceed with support regardless.” These examples illustrate how learning was not theoretical but activated through concrete, often emotionally charged, workplace events.

#### From reactive to reflective - rethinking organisational learning

A recurrent insight was that policies and procedures tend to remain dormant until activated by crises. As one participant put it, “Only when faced with real cases did routines embed within our organisation.” This reactive pattern was widely recognised as problematic, pointing to a need for more proactive and continuous learning structures. The lab thus encouraged participants to shift from crisis-driven responses to a reflective learning culture - where alcohol prevention is not an isolated task but integrated into the organisational fabric. “Long-term employees may overlook gradual changes - are we adapting adequately?” one participant asked, underscoring the need for ongoing organisational self-awareness.

#### Summary - a call for dialogue, structure, and cultural change

Learning Lab 1 revealed how effective alcohol prevention cannot rest solely on policy, awareness, or personal motivation. Instead, it requires sustained dialogue, emotionally intelligent leadership, and supportive organisational structures. Participants advocated for environments where conversations about alcohol and health are not taboo but part of everyday leadership practice. In doing so, the lab marked a shift, from viewing alcohol-related issues as isolated incidents to seeing them as indicators of broader organisational dynamics, cultural norms, and systemic responsibilities. Through dialogical, emotional, and practice-based learning, participants took steps in their cognitive, emotional, cultural and structural understanding toward building workplaces that are both responsive and resilient.

### Learning lab 2: from awareness to strategic understanding

As the process moved into Learning Lab 2, the focus expanded from emotional resonance and shared reflection toward a more conceptual and strategic understanding of alcohol prevention. Participants returned with renewed perspectives, some with deepened roles, others with fresh organisational experiences. The session was built on the collective foundation of Learning Lab 1 but introduced new results of the survey and structured, research-based knowledge that reframed how participants conceptualised prevention work within their organisations.

#### Reframing prevention - integrating theory and practice

The session began with participants raising critical questions: *How do we move alcohol prevention from the margins to the core of organisational responsibility?* Discussions quickly moved beyond surface-level policy concerns, emphasising the need to embed alcohol prevention within the broader frameworks of sustainable leadership, workplace safety, and employee well-being. Researchers contributed theoretical frameworks such as universal, selective, and indicated prevention models, which helped participants reassess their existing assumptions. For many, this shift was transformative. One participant shared that the researcher’s way of defining prevention was new to her, suggesting that theoretical input opened new avenues of thought. However, the integration of theory into lived organisational realities revealed tensions. As another participant remarked, *“The challenge with universal strategies is that they don’t really touch the heart.”* The lab thus highlighted the importance of aligning cognitive understanding with emotional and cultural engagement for prevention to be truly effective.

#### The policy-culture gap - “Just words if you don’t dare to talk about it”

A core insight from Learning Lab 2 was the persistent disconnect between formal alcohol policies and everyday workplace culture. While many organisations had policies in place, participants described how these often remained symbolic - lacking traction in practice. *“We may have the world’s best policy*,* but it’s just words if you don’t dare to talk about it…”* This statement captured the gap between structural intent and lived experience.

This disconnect was most apparent in what participants described as a “lost middle ground” - a space between vague concern and formal intervention. As one person observed, *“There’s a middle ground between having a policy and entering treatment…”* Participants called for intermediate strategies that allow for early conversations and low-threshold support before crises emerge.

#### Mandates, managerial uncertainty, and relational leadership

The lab also surfaced learning around unclear mandates and managerial hesitation. Some participants openly questioned whether preventive work around alcohol even fell within their role. This hesitation underscored how ambiguity around responsibility can undermine responsiveness, allowing tolerance to persist by default. At the same time, participants reflected on the powerful role of leadership as cultural signalling. *“Those with formal mandates must also live by the values…”* one participant noted, emphasising that leadership is not only about policy enforcement but about role modelling and trust-building. Informal leadership also surfaced as crucial, cultural change often followed informal norms more than formal declarations.

#### Alcohol as a health issue - toward cultural normalisation

One of the most striking insights was the difficulty of integrating alcohol into broader workplace health conversations. While topics like stress and sleep were commonly discussed in employee dialogues, alcohol remained taboo. As one participant questioned, *“We ask about stress and sleep in employee dialogues*,* but would we ever ask about alcohol?”* This rhetorical question highlighted persistent stigma, where alcohol remains culturally separate from mainstream health narratives. Participants agreed that reframing alcohol prevention within wellness-oriented language and initiatives could be more effective than standalone control strategies. *“Framing alcohol prevention within an overall wellness perspective can create a more sustainable and effective strategy*,*”* one concluded.

#### Structural fragility and organisational learning

Participants also noted that preventive efforts often depend on individual initiative rather than embedded routines. One participant described how external partnerships and structured processes had created stability - only to be disrupted by organisational change: *“After reorganisation*,* we lost that structure. Now there’s uncertainty.”* This experience pointed to the fragility of prevention efforts when not institutionalised. The lab emphasised the importance of continuous learning spaces to bridge the gap between policy and daily routines. As one participant asked, *“We have policies*,* but how do we make them an integral part of daily routines?”* Informal norms, emotional safety, and clarity of responsibility were all identified as critical factors for sustainable prevention work.

#### Learning processes and emerging strategies

Throughout Learning Lab 2, participants identified key areas of learning: (a) Cognitive and conceptual learning emerged from engaging with theoretical models that helped reframe organisational responsibilities. (b) Cultural learning involved recognising the stigmas and silences that prevent open conversations around alcohol. (c) Strategic learning was activated as participants considered how to bridge the gap between documentation and lived practice. Participants also reflected on how alcohol prevention must be embedded in broader systems of organisational care, not treated as a separate or exceptional topic. This included recognising the need for more structured collaboration with external health actors, clearer role definitions, and everyday leadership anchored in trust and openness.

#### Summary - toward cultural and structural integration

Learning Lab 2 functioned not merely as a forum for knowledge exchange, but as a collective site of cultural critique and strategic development. It revealed that even in the presence of well-developed policies, meaningful prevention requires emotionally resonant, socially embedded, and structurally supported processes. Participants voiced a shared desire to continue these conversations beyond the lab: *“Continuing what we did today would be very rewarding.”* In short, Learning Lab 2 deepened the collective understanding of alcohol prevention, not as a narrow or disciplinary task, but as a complex organisational practice that bridges theory and culture, policy and action, care and responsibility.

### Learning lab 3: from insight to action

By the third and final Learning Lab, a clear developmental arc had emerged. Participants demonstrated a growing confidence in integrating alcohol prevention into broader workplace wellness efforts. The sustained process, marked by dialogue, shared experience, and repeated theoretical inputs, culminated in a phase of applied learning, where previously abstract and emotional insights began translating into concrete strategies and organisational tools.

#### The role of the survey as structured knowledge input

During Learning Lab 3, a researcher from the team presented survey results on managers’ perceptions of their formal responsibility to engage in alcohol prevention efforts, including gender-disaggregated statistics on the extent to which they felt accountable for such work. This was followed by data showing, among those who reported feeling a sense of responsibility, the distribution of responses concerning whether they felt they had sufficient knowledge, felt confident in supporting employees with alcohol-related issues, believed they could access support, felt that workplace guidelines and occupational health efforts provided adequate support, and whether they perceived that employees accepted managers addressing alcohol prevention topics. The same set of results was then presented for the managers who stated they did not consider themselves responsible for engaging in alcohol prevention work. Following the presentation, participants were encouraged to reflect on the findings they had just reviewed.

#### Embracing preventive responsibility as a core leadership practice

One of the strongest signals of learning in Lab 3 was the reframing of preventive responsibility. No longer seen as an isolated duty, alcohol prevention was increasingly understood as an integral part of sustainable leadership and general health promotion.

As one participant reflected, *“I definitely think we have a preventive responsibility […] it’s about general ill-health*,* where I think the alcohol question comes in.”* This quote captures the deepened awareness that had emerged over the course of the labs, positioning alcohol not as a standalone risk factor but as part of a larger pattern of workplace well-being.

#### From concept to method - the role of tools in bridging the gap

A significant shift occurred as participants began highlighting the value of method-driven approaches, including specific tools like the *Health Talk Tool* and the *BAT-tool*. These instruments were not seen as generic checklists but as practical enablers of preventive dialogue. One participant shared, *“For several years*,* we’ve had a tool called Health Talk Tool […] intended to be used perhaps twice a year.”* Such tools were recognised as a way to systematically introduce sensitive topics into routine conversations, thereby normalising them and lowering thresholds for action. This phase of learning was marked by a clear movement from conceptual frameworks to applied organisational practices, where theory became embedded in routines and structures.

#### Dialogue, interaction, and shared understanding

Participants described the lab as a space for collective reflection, one that enabled learning to emerge dialogically, rather than through isolated thinking. “*The interactively in the learning lab*,* it’s complete group learning*,” one participant explained. Another reflected, *“When working interactively in the learning lab*,* the group’s learning becomes total*.” These comments underscore the centrality of a co-constructed process where knowledge was formed through the convergence of multiple perspectives. The format fostered openness and curiosity. “*We just ask a question*,* and then we can talk for ages*,*”* one participant noted, capturing the organic, evolving nature of the discussions. This dialogical space allowed professionals to reflect on ambiguity, uncertainty, and complexity - elements often absent from traditional training environments.

#### Emotional engagement and practical anchoring

Participants emphasised the importance of real-life cases, which brought emotional and moral dimensions into the learning process. “*They worked on a real case*…,” one participant noted, highlighting how practical relevance enhanced the applicability of the learning. These grounded discussions invited participants to connect with emotionally charged realities they often face in their roles. One participant stated, “*Realising that I can do it with care*…,” pointing to how empathy and ethical commitment shaped how they thought about intervention. The participant continued, “*It’s hard to get an addiction diagnosis if the person doesn’t admit it*…,” a comment that speaks to the real-world tensions between formal criteria and human complexity.

#### Learning through ntegration - combining research and practice

Participants also emphasised the enriched learning experience created by combining theoretical inputs with dialogue and reflection. One participant remarked, *”What’s great is that we’re sitting here reflecting on how we perceive and work with these issues in practice […] And then you’ve combined this with ongoing research […] This makes our conversation much richer […] multilayered in a way I’ve really appreciated.”* This comment highlights how theory was not experienced as top-down instruction but as a catalyst for collaborative inquiry, enabling participants to reframe their experiences in new and more strategic ways.

#### Responsiveness and the ongoing challenge of tolerance

Preventive action was closely tied to the theme of responsiveness, with participants identifying dialogue and training as critical tools. *“It’s the dialogue itself that’s important*,* not how you rate yourself*,*”* one participant emphasised. Dialogue was no longer seen as vague or abstract, it had become a primary tool for early detection and care. In contrast, tolerance emerged as a function of organisational inertia and managerial insecurity. One participant observed, *“Managers are very insecure when it comes to alcohol-related issues. They don’t even dare to ask employees if they have a problem with alcohol.”* This insight underscored that passivity is not rooted in indifference, but in fear, lack of training, and unclear mandates, highlighting the ongoing need for structural and cultural support.

#### Sustained engagement and collective reflection

The third lab also functioned as a space for meta-reflection, where participants evaluated the learning process itself. Many spoke of the importance of exchanging experiences with peers from other organisations, *“I find it very interesting meeting representatives from other companies. After all*,* we share the same challenges.”* Others acknowledged the value of recurring reminders, *“This reminds us again*,* ‘Oh wow*,* this issue isn’t done. We might never fully resolve it.’* Such reflections suggest that participants had embraced a mindset of ongoing learning, recognising prevention not as a finite task but as a continuous organisational process.

#### Desires for further development - depth, frequency, and shared ownership

Participants concluded the lab by voicing suggestions for improvement and further development. One pointed to the long gaps between sessions, *“Perhaps the intervals have been too long. I believe shorter intervals would have been better.”* Another advocated for bringing more colleagues into the knowledge integrated learning process to strengthen the Learning Labs, *“Maybe it could be an idea to have at least two people from the same organisation. That way*,* when you return*,* you have shared experiences and can continue to discuss with each other.”* This suggestion reflects a growing recognition of the need for shared ownership and internal dissemination of learning within organisations.

There was also a call for more concrete, real-life case studies to guide implementation. This underscores a desire to translate reflective learning into practical action, and to have more tangible templates for applying insights.

#### Summary - toward sustainable, embedded prevention

Learning Lab 3 marked a shift from reflection to action. Participants expressed a clear understanding that sustainable alcohol prevention requires: (a) Clarity in managerial roles and mandates, (b) Systematic support through practical tools, (c) Integration of alcohol prevention into general wellness dialogues, and (d) Cultural normalisation through regular, supported conversation. By the end of the process, participants no longer framed alcohol prevention as an isolated concern or compliance issue. Instead, they saw it as part of an ongoing organisational commitment to health, trust, and psychological safety. As one participant concluded, the labs had helped them move from fragmented understanding to a more holistic, actionable strategy, anchored in dialogue, shared responsibility, and preventive structure.

## Discussion

The learning labs illustrated a progressive development in how HR professionals perceived and made sense of alcohol prevention in their organisations. Initially grounded in emotional and experiential learning, participants confronted cultural and structural barriers such as stigma and ambiguity around responsibility. Over time, theoretical input supported a reframing of assumptions, highlighting gaps between policy and practice. By the final lab, participants described how they had begun to conceptualise more concrete ideas about preventive routines and leadership support. Importantly, these developments reflect changes in participants’ understanding, not necessarily changes in organisational practice itself.

This developmental trajectory aligns with prior research showing that multi-component and participatory learning approaches can strengthen preventive capacity in workplace health interventions [3, 7, 19]. Moreover, the iterative structure of the learning labs shows how the continuous integration of theoretical and practical knowledge can support cognitive and conceptual shifts, an insight consistent with research on organisational learning and workplace interventions [9, 24].

The progression across the three Learning Labs is reflected in several distinct analytical perspectives:

### Dialogue-based and co-created knowledge

Participants consistently emphasised the value of collective reflection. This aligns with earlier findings [5, 19] showing that dialogical processes can deepen learning and foster shared understanding. In our study, dialogue created the conditions for HR professionals to articulate uncertainties, challenge assumptions, and begin reframing alcohol prevention as part of everyday leadership work.

###  Experience-based learning and practical dilemmas

Emotionally charged, real-life cases were powerful triggers for reflection. These cases grounded theoretical and policy discussions in the lived realities of HR work, underscoring the importance of contextualised learning consistent with Arends et al. [3].

###  Emotional and cultural barriers to action

Participants often described how discomfort, stigma, and organisational norms inhibited preventive action. These findings support earlier research [9, 18], indicating that cultural barriers must be addressed for responsiveness to emerge.

###  Structural ambiguity and unclear mandates

Ambiguity around roles and responsibilities surfaced as a key obstacle. Participants repeatedly emphasised the need for clearer mandates, better-defined expectations, and embedded routines, findings aligned with Haslam et al. [15] and Daniels et al. [7]. These insights also reflect the distinctive position of HR in Nordic organisations, where formal systems intersect with culturally shaped leadership expectations [14, 17].

###  Managerial insecurity and the role of dialogue

Participants described managerial insecurity as a major reason for organisational passivity. Strengthening dialogue and creating a supportive culture were seen as essential for responsiveness, echoing Sorensen et al. [24].

Taken together, these perspectives form an integrated conceptual model consisting of three interconnected dimensions:

- collective and dialogical knowledge creation;
- emotional and cultural awareness; and
- structurally anchored mandates and routines.

This model conceptualises alcohol-preventive capacity not as a single skill or policy, but as a situated interplay between HR sensemaking, organisational norms, and available structures.

This integrated perspective extends earlier frameworks [1, 8, 19] by delineating how emotional, cultural, structural, and dialogical factors interact in shaping preventive readiness. Antonacopoulou’s concept of practising [2] further supports this interpretation, as participants engaged in cycles of reflection and re-evaluation that reshaped their understanding of alcohol norms and preventive responsibility. This also mirrors the survey findings, where gaps between attitudes and action became key learning triggers. The combination of theoretical knowledge and practice-based reflection was repeatedly identified by participants as central to learning, aligning with Langley et al. [20] and Daniels et al. [7].

Importantly, participants’ understanding of tolerance and responsiveness evolved throughout the labs. What began as emotionally anchored, interpersonal concerns increasingly came to be seen as embedded in broader cultural and structural conditions as perceived by HR participants. These shifts represent changes in HR professionals’ preventive reasoning, not verified organisational change.

In sum, the enhanced understanding observed in the labs resulted from the iterative interplay among collective reflection, structured knowledge input, emotional engagement, and practical anchoring. This design enabled participants to move from fragmented, reactive perspectives toward more coherent and situated ways of conceptualising prevention. While we cannot determine whether participants implemented new practices, the study demonstrates how integrated learning processes can strengthen preventive awareness and perceived organisational readiness.

Practically, the findings suggest that effective alcohol prevention strategies must be embedded in ongoing learning processes and supported by clear mandates, leadership engagement, and structured tools. Organisations can benefit from creating cultures that reduce managerial uncertainty, support open dialogue, and integrate alcohol prevention into broader wellness and safety frameworks. This aligns with multi-component strategies proposed by Elling et al. [8] and Sorensen et al. [24].

At the same time, several limitations must be acknowledged. The learning labs constituted safe, facilitated environments, which may have encouraged idealised or aspirational accounts. The findings represent HR professionals’ perspectives and sensemaking, which may differ from line managers’ lived experiences. This reflects a known gap between HR-level intentions and managerial enactment. Furthermore, having only one or two HR representatives per organisation limited internal knowledge diffusion and anchoring. A more frequent or expanded format might have supported deeper organisational integration. Finally, the absence of line managers in the study prevented triangulation of perspectives; future research should include managerial and employee-level data to capture how preventive intentions translate into practice.

## Conclusion

 This study explored how a knowledge-integrated learning process can reduce organisational tolerance toward risky alcohol use and enhance preventive responsiveness. The findings demonstrate that sustained, participatory learning, combining emotional engagement, conceptual input, and practical tools, supported HR professionals in reframing alcohol prevention as they perceived it, rather than indicating verified organisational change. Through the identification of five interlinked mechanisms, dialogue-based learning, experience-based reflection, emotional and cultural barriers, structural ambiguity, and managerial insecurity, the study highlights how participants’ understanding of prevention evolved when knowledge was co-created and shared across roles. Rather than suggesting verified organisational change, the findings illustrate shifts in how HR professionals conceptualised their preventive role.

 Practically, the results suggest that effective workplace alcohol prevention requires clear mandates, supportive leadership, and continuous dialogue - embedded not as a one-off initiative but within ongoing organisational learning processes. While the findings are context-specific and derived from HR perspectives, the knowledge-integrated learning model offers a conceptually transferable framework for improving workplace health and responsiveness.

### Limitations and future research

 This study’s findings are shaped by its qualitative design and context within large Swedish organisations, which may limit transferability. The sample consisted primarily of HR representatives, and perspectives from line managers or employees were not included. As a result, the study reflects HR professionals’ sensemaking rather than verified organisational practice. In addition, having only one or two participants per organisation constrained internal knowledge diffusion and organisational anchoring.

 Future research should test the proposed model in diverse organisational and cultural settings, ideally through larger mixed-method or quantitative studies. Longitudinal research examining how knowledge-integrated learning processes translate into actual cultural and structural change would also be valuable. Expanding this work into related areas of substance use and organisational well-being may further strengthen the empirical foundations of integrative, learning-oriented prevention frameworks.

 In summary, this study provides a nuanced theoretical and practical understanding of workplace alcohol prevention by demonstrating how collective, dialogical, emotional, and structural learning processes shape preventive reasoning within organisations. Such integrative approaches hold significant potential for fostering responsive, sustainable, and context-sensitive prevention strategies in a wide range of workplace settings.

## Data Availability

The datasets generated or analysed during this study are not publicly available due to confidentiality and ethical restrictions. Anonymised excerpts relevant to the findings are included in this article. Additional data may be available from the corresponding author upon reasonable request and subject to Ethics Review Board approval.

## Abbreviations

BAT-tool: Brief assessment tool (workplace screening instrument)
EAP: Employee assistance programs
HLV: Health on equal terms (national Swedish public health survey)
HR: Human resources
